# Supplementary material for: Prevalence of hypertension and factors associated with the utilization of primary health care services for hypertension among hypertensive population aged 40 years and above in Pyin Oo Lwin Township, Myanmar
Source: PLoS One. 2024 Oct 16;19(10):e0312186. doi: 10.1371/journal.pone.0312186 (PMC11482684; doi:10.1371/journal.pone.0312186)
Supplement: S1 File — (DOCX) [file pone.0312186.s001.docx]

**List of Abbreviations**

PEN - Package of essential non-communicable diseases intervention

JNC7 - The seven^th^ Joint National Committee

NCDs - non-communicable diseases

CVD - cardiovascular diseases

PHC - Primary health care

OOP - Out-of-pocket

BHS - Basic health staff

MCH - Maternal and child health care center

RHC - Rural health center

Sub-RHC - Sub rural health center

UHC - Urban health center

BP - Blood pressure

AHA - American Heart Association

LHV - Lady health visitor

SEAR - South-East Asia Region
