# Supplementary material for: Prevalence of hypertension and factors associated with the utilization of primary health care services for hypertension among hypertensive population aged 40 years and above in Pyin Oo Lwin Township, Myanmar
Source: PLoS One. 2024 Oct 16;19(10):e0312186. doi: 10.1371/journal.pone.0312186 (PMC11482684; doi:10.1371/journal.pone.0312186)
Supplement: S1 Table — (DOCX) [file pone.0312186.s002.docx]

**S1 Table. Bivariate analysis between independent variables and the utilization of PHC Services among the hypertensive study participants (n=386)**

| Variables | Class | Hypertension  (n=386) | Utilization | | p-value |
| --- | --- | --- | --- | --- | --- |
|  |  |  | **Yes (n = 200)** | **No (n = 186)** |  |
| Age group (years) | 40-49 | 76 (19.7%) | 33 (43%) | 43 (57%) | 0.228 |
|  | 50-59 | 110 (28.5%) | 57 (52%) | 53 (48%) |  |
|  | ≥60 | 200 (51.8%) | 110 (55%) | 90 (45%) |  |
| Gender | Male | 125 (32.3%) | 60 (48.0%) | 65 (52.0%) | 0.353 |
|  | Female | 261 (67.7%) | 140 (53.6%) | 121 (46.4%) |  |
| Residence | Urban | 128 (33.2%) | 44 (34%) | 84 (66%) | **<0.001** |
|  | Rural | 258 (66.8%) | 156 (60%) | 102 (40%) |  |
| Marriage | Not currently married | 135 (34.9%) | 65 (48.2%) | 70 (51.8%) | 0.342 |
|  | Currently married | 251 (65.1%) | 135 (53.8%) | 116 (46.2%) |  |
| Education | Below High school | 352 (91.1%) | 189 (53.7%) | 163 (46.3%) | **0.028** |
|  | High school and above | 34 (8.9%) | 11 (32.4%) | 23 (67.6%) |  |
| Occupation | Employed | 218 (56.5%) | 109 (50%) | 109 (50%) | 0.478 |
|  | Unemployed | 168 (43.5%) | 91 (54.2%) | 77 (45.8%) |  |
| Monthly family income | ≤150,000 | 103 (28.5%) | 45 (43.7%) | 58 (56.3%) | 0.110 |
| (MMK) (n= 362) | >150,000 | 259 (71.5%) | 139 (53.7%) | 120 (46.3%) |  |
| Ethnicity | Burmese | 312 (80.8%) | 161 (51.6%) | 151 (48.4%) | 0.967 |
|  | Others | 74 (19.2%) | 39 (52.7%) | 35 (47.3%) |  |
| Religion | Buddhist | 374(96.9%) | 180 (96.8%) | 194 (97.0%) | 0.898 |
|  | Others | 12 (3.1%) | 6 (3.2%) | 6 (3.0%) |  |
| Number of family members | 1-4 | 225 (58.3%) | 108 (48%) | 117 (52.0%) | 0.095 |
|  | >4 | 161 (41.7%) | 92 (57.1%) | 69 (42.9%) |  |
| Family history of HTN | Present | 161 (41.7%) | 82 (50.9%) | 79 (49.1%) | 0.849 |
|  | Others | 225 (58.3%) | 118 (52.4%) | 107 (47.6%) |  |
| Social or financial support | Yes | 251 (65.0%) | 147 (58.6%) | 104 (41.4%) | **<0.001** |
|  | No | 135 (35.0%) | 53(39.3%) | 82(60.7%) |  |
| Known status of HTN | Known | 306 (79.3%) | 23 (28.7%) | 57 (71.3%) | **<0.001** |
|  | Unknown | 80 (20.7%) | 177 (57.8%) | 129 (42.2%) |  |
| Perceived health status | Good | 238 (61.6%) | 118 (49.6%) | 120 (50.4%) | 0.313 |
|  | Poor | 148 (38.4%) | 82 (55.4%) | 66 (44.6%) |  |
| Number of comorbidities | None | 202 (52.3%) | 98 (48.5%) | 104 (51.5%) | 0.397 |
|  | At least one | 132 (34.2%) | 73 (55.3%) | 59 (44.7%) |  |
|  | 2 and above | 52 (13.5%) | 29 (55.8%) | 23 (44.2%) |  |
| Level of knowledge | Low | 244 (63.2%) | 127 (52.1%) | 117 (47.9%) | 0.987 |
|  | Average/High | 142 (36.8%) | 73 (51.4%) | 69 (48.6%) |  |
| Perception on HTN | Poor/Average | 340 (88.1%) | 185 (54.4%) | 155 (45.6%) | **0.009** |
|  | Good | 46 (11.9%) | 15 (32.6%) | 31 (67.4%) |  |
| PHC facility | Absent | 191(49.5%) | 81 (42.4%) | 110 (57.6%) | **<0.001** |
|  | Present | 195 (50.5%) | 119 (61.1%) | 76 (38.9%) |  |
| Travel time | <30min | 353 (91.5%) | 183 (51.8%) | 170 (48.2%) | 1.000 |
|  | >30min | 33 (8.5%) | 17 (51.5%) | 16 (48.5%) |  |
| Perceived cost of travel | Not necessary | 219 (56.7%) | 128 (58.5%) | 91 (41.5%) | **0.0039** |
|  | Cost | 167 (43.3%) | 72 (43.1%) | 95 (56.9%) |  |
| Awareness of available services | Aware | 207 (53.6%) | 138 (66.7%) | 69 (33.3%) | **<0.001** |
|  | Not aware | 179 (43.4%) | 62 (34.6%) | 117 (65.4%) |  |
| Convenience with clinic hour | Yes | 386 (100%) | 200 (51.8%) | 186 (48.2%) | 1.000* |
|  | No | 0 (0%) | 0 (0%) | 0 (0%) |  |
| Perception on public health facilities | Poor/Average | 367 (95.1%) | 189 (51.5%) | 178 (48.5%) | 0.7576 |
|  | Good | 19 (4.9%) | 11 (57.9%) | 8 (42.1%) |  |

* P values are from Fisher exact test
