# Supplementary material for: Prevalence of hypertension and factors associated with the utilization of primary health care services for hypertension among hypertensive population aged 40 years and above in Pyin Oo Lwin Township, Myanmar
Source: PLoS One. 2024 Oct 16;19(10):e0312186. doi: 10.1371/journal.pone.0312186 (PMC11482684; doi:10.1371/journal.pone.0312186)
Supplement: S2 Table — (DOCX) [file pone.0312186.s003.docx]

**S2 Table. Factors associated with the utilization of PHC Services by multiple logistic regression models**

| Variable | | Full model ^ǂ^ | | Final model^€^ | |
| --- | --- | --- | --- | --- | --- |
|  |  | **AOR** | **95%CI** | **AOR** | **95% CI** |
| Place of Residence | Urban | 1 |  | **1** |  |
|  | Rural | 2.51*** | 1.47, 4.34 | **2.79***** | **1.68, 4.67** |
| Education | <High school | 1 |  |  |  |
|  | ≥High school | 0.49 | 0.20, 1.17 |  |  |
| Monthly Family Income (n=362) | ≤150,000 MMK | 1 |  |  |  |
|  | >150,000 MMK | 1.40 | 0.81, 2.42 |  |  |
| Number of Family member | 1 - 4 | 1 |  |  |  |
|  | >4 | 1.40 | 0.85, 2.31 |  |  |
| Financial/Social Support | No | 1 |  |  |  |
|  | Yes | 1.19 | 0.70, 2.02 |  |  |
| Status of HTN | Unknown | 1 |  | **1** |  |
|  | Known | 4.17*** | 2.23, 8.07 | **4.36***** | **2.39, 8.23** |
| Perception towards HTN | Low/Fair | 1 |  | **1** |  |
|  | High | 0.35** | 0.16, 0.75 | **0.30**** | **0.14, 0.62** |
| PHC Facility | Absent | 1 |  |  |  |
|  | Present | 1.46 | 0.88, 2.41 |  |  |
| Perceived Cost of Travel | Not necessary | 1 |  | **1** |  |
|  | Cost | 0.60* | 0.36, 0.98 | **0.57*** | **0.35, 0.92** |
| Awareness of Services | Not aware | 1 |  | **1** |  |
|  | Aware | 3.86*** | 2.37, 6.38 | **4.11***** | **2.55, 6.71** |
| Observations | | 362 |  | 362 |  |
| Hosmer-Lemeshow goodness-of-fit χ^2^ (df) | | 16.86 | (8) | 7.68 | (7) |
| P value for HL χ^2^test | | 0.031 |  | 0.362 |  |
| AIC* | | 419.5 |  | 418.5 |  |

*****Akaike information criterion
