# Supplementary material for: Prevalence of hypertension and factors associated with the utilization of primary health care services for hypertension among hypertensive population aged 40 years and above in Pyin Oo Lwin Township, Myanmar
Source: PLoS One. 2024 Oct 16;19(10):e0312186. doi: 10.1371/journal.pone.0312186 (PMC11482684; doi:10.1371/journal.pone.0312186)
Supplement: Attachment — Submitted filename: Response to Reviewers.docx [file pone.0312186.s005.docx]

 Dear Sayar Show,

Thank you very much for the comments of the reviewers and the editor. We have addressed all these comments and given a point-by-point response below, using **bold font**.

**Reviewer #1:**

**We thank the reviewer for giving comments and pointing out some points which need clarification. We respond as below: -**

1. The abstract's methods section briefly describes the study design but lacks important details like how participants were selected and the response rate.

**We have revised the methodology section of the abstract with brief description of sampling method, response rate and the classification of blood pressure for hypertension.**

2. The rationale for determining the sample size is well-explained, considering factors like confidence interval and margin of error. However, there's a discrepancy between the reported prevalence rates (88% vs. 28.9% from national estimates). To ensure accuracy, it's important to clarify how these differences were accounted for in the sample size calculation.

**We have made those two prevalence rates clear in revision that the prevalence of hypertension among population was 28.9% and the prevalence of hypertensive individuals who utilized primary health care facilities was 88%.**

3. The sampling procedure should address any potential biases introduced during participant selection.

**We have discussed potential biases in the revised manuscript.**

4. The definition of primary health care service utilization for hypertension is clearly defined, covering different types of health facilities and services. To strengthen this section, it would be beneficial to provide more details or validation methods on how utilization was measured in the health system.

**We have mentioned details about validation methods for outcome measures as you suggested.**

5. It would be helpful to briefly mention any steps taken to protect participant confidentiality and ensure data security during and after data collection.

**We have added information about what we did for maintaining participant confidentiality in methodology section.**

6. It would be good to clarify whether the study used age-standardization when comparing hypertension prevalence rates with other studies.

**We have compared this study prevalence with other studies prevalence rates after age-standardization. We have put this information in the revised manuscript.**

7. It would be better to expand on how these findings could inform the improvements of broader health policy and primary healthcare system in Myanmar.

**We have discussed some policy implications that would assist in strengthening of primary health system in discussion section.**

8. Comparing findings with studies from other countries adds depth to the discussion. However, it's important to clearly explain how these comparisons were made, considering differences in healthcare systems and socio-economic factors.

**In the revised manuscript, we have made prevalence comparison with other Low-and-Middle income countries from South-East Asia with similar socioeconomic backgrounds. So, we have removed some prevalence rates of the countries which was mentioned in the previous manuscript, because of different socio-economic and geographical backgrounds (China, India and Nigeria) in making comparison.**

**Reviewer #2:**

**We thank you the reviewer for insightful comments. We responded as below: -**

In Financial Disclosure, it is mentioned that “The author(s) received no specific funding for this work”. However, the authors acknowledged the IR grant committee of Department of Medical Research, Ministry of Health. Could you please clarify whether this study was funded by the grant from IR grant committee of Department of Medical Research, Ministry of Health? If not, could you explain how the expenses for this study were covered.

**We have got the IR grant from IR grant committee of Department of Medical Research, Ministry of Health. It covered for the expenses of data collection, but did not cover for the work of publication.**

I would like to suggest a through proofread. Some sentences are lengthy and could be rephrased for better clarity and readability.

**Yes. We have modified some lengthy sentences into readable ones in the revised manuscript.**

The abstract should serve as a standalone summary, including all essential details for comprehension without referring to the full manuscript. In this abstract, methods need to be elaborated further, and results and conclusion should be aligned.

**We have made sure this revised abstract is concise, complete and comprehending. We also have revised the methodology section to be complete, and results and conclusion sections to be aligned.**

**We also have comments from another reviewer for the point of results not being aligned with conclusion.**

**We have modified it and discussed it in discussion section of the revised manuscript.**

Line 29: Please specify the year in which stroke was the first leading cause of death in Myanmar

**It was the result of Verbal autopsy survey 2014-2016.**

In Introduction section, it would be better to explain the reasons for choosing Pyin Oo Lwin Township and the population aged 40 years and above. Additionally, please clarify whether PHC services users need to pay for consultation fees or medicine.

**According to PEN guidelines, 40 years and above is the target age group and it is considered as risk in calculating cardiovascular risk scores.**

**After PEN projects had piloted two townships of Yangon in 2012, PEN was expanded in 20 townships of 5 States and Regions in 2017. Pyin Oo Lwin was included in not only the extended townships, but also, piloted townships for Mandalay Region.**

**In Myanmar Health Statistics 2019, it mentioned Mandalay was one of the Regions with higher prevalences of hypertension. Besides, whether hypertension services (including screening and treatment) provided at public health facilities of Pyin Oo Lwin Township were underutilized or not, was uncertain. This may be due to increased screening activities of the Region.**

**However, number of people who utilize the services was much lower than that of 40 years and above township population according to Pyin Oo Lwin Township Public Health Department. That’s why we chose the study population was 40 years and above population of Pyin Oo Lwin Township.**

**Besides, PHC services provided at public health facilities do not need to pay for consultation fees and essential medicine if they are not stock-out. However, whenever there is shortage of medicines supply in health facilities, patients have to purchase medicines and medical supplies at retail pharmacies. costs patients with out-of-pocket expenditures.**

Line 51-59: it is better to provide descriptions of other PHC services providers in study site such as GP clinics, or outpatient departments which are not under the township public health department and should explain the reasons why these are not included as PHC services providers in this study.

**This study did not cover private PHC facilities including GPs. So, we have discussed it in both methodology and discussion sections of the revised manuscript.**

Line 71-79: It would be helpful to provide description of the location of the 7 villages and 3 wards.

**We have added some information regarding the description of location of the study site in methodology section.**

Line 42: Please correct the grammar for “PHC-oriented research were needed”

Line 98: “Data was collected” should be “Data were collected”

Line 104: “two-parts” should be “two parts”

Line 161: Please clarify “not aware of their statuses”. I assume “not aware of their hypertension”.

Line 168: “Table (1) showed” should be “Table (1) shows”

Line 181: “Shown in figure (2)” should be “As shown in figure (2)”.

Line 195: “Age composition” should be “The age composition” checked

Line 199: “30-79Years” should be “30-79 years” checked

Line 270: I would like to suggest “economic inaccessibility” instead of “economic accessibility” checked

**Those mistakes have been corrected.**

Line 173: Could you please clarify the rationale for using 150,000 MMK as the cutoff point?

**Until October 2023, Daily wages of a person was 4800 MMK. At least for single income households, they should be earned 150,000 MMK per month. It was similar to the income levels of Household Amenities in Myanmar (2014 - 2019), nearly over one third of households lived with annual income of 1.8 million MMK which was 150,000 MMK.**

Line 185-186: Please clarify high perception and low perception.

**For better clarification, we have changed the terms “high” and “low” into “good” and “poor” perception.**

**In this study, perception on hypertension is meant for a person’s own view or interpretation of control of hypertension. Perception on health facilities is meant for a person’s own view or interpretation that he or she has about public PHC facilities; how they stand in the community.** **Both perceptions are classified as poor (<50% scores), average (50-75% scores) and good (>75% scores).**

In Results section, it would be better to include relevant findings from bivariate analysis. Additionally, important negative findings-factors not associated with utilization of PHC services-should also be described and discussed in the main manuscript.

**We have discussed associations of outcome measures with some relevant findings in descriptive and bivariate analyses though they are not significant predictors in multivariate analysis.**

Line 207: Can you please spell out “SEAR”

**SEAR stands for South-East Asia Region. We have described it in abbreviations. We have also clarified it in the main manuscript.**

Line 220: Please spell out LHV

**LHV stands for Lady Health Visitor. It has also been described in abbreviations. We have also clarified it in the main manuscript.**

Line 226: I would like to suggest using “However” instead of “But” at the beginning of the sentence.

**We have modified it according to your suggestion.**

Line 248-249: Could you please elaborate more on “Consequently, urban residents choose health facilities based on their conveniences.”

**We have modified that sentence in the revised manuscript.**

**Secondary and tertiary public hospitals are mainly located in urban. Private health facilities (Private hospitals, Specialist clinics and GP clinics) are more condensed in urban. At the same time, urban had lower number of public primary health facilities than rural. Public hospitals are crowded and ambulatory OPD are not convenient with their working time. In addition, Myanmar has not established the mandatory tiered referral system.**

**At that time, urban residents choose health facilities based on their conveniences; which have geographical accessibility, affordability, sensitivity to their needs** **that meet to their satisfaction.**

In Discussion section: It would be better to explore the possible reasons behind the fact that over two third of the participants were female, as well as operation hours of the PHC service centers. Discussing the implication of these findings and comparisons with other studies could be beneficial. For instance, consider addressing how these differences or similarities affect our understanding of hypertension prevalence, management and PHC utilization in your study population. Furthermore, discussing potential strategies, policy recommendation, and areas for further research to improve the situation would be valuable.

**We have ensured that, as a community-based cross-sectional study conducting in day time, male population are less likely to stay at home and participate in the study, unless we made the selection bias controlled; it was a limitation of the study. We mentioned other coinciding studies with references in the discussion section.**

**We thought the overall prevalence may have biased in any directions but, in Myanmar, male have higher behavioral risk factors than female. So, we inferred that low participation of male population may bring about underestimation of the overall prevalence.**

**We have recommended to prioritize NCDs care for economically active population at primary health care level for early detection and timely treatment.**

Line 296-298: Could you please provide more details on how the findings from this study support the idea that quality assurance of PHC services improves the utilization of these services for hypertension.

**Thank you for pointing that out. We have this comment from another reviewer as well.**

**Our study assessed the utilization of PHC services for hypertension. We found the utilization rate was low and we have discussed the possible reasons for low utilization and included about health seeking at higher quality of care and discussed it with references.**

**Moreover, we have also found that participants with good perception on hypertension and higher education were less likely to utilize the public health facilities.**

**Kujawski et al. explored reasons for low utilization of public health facilities. In that study, households with hypertension tended to utilize private healthcare at facilities staffed with qualified providers.**

**That study also found that choosing private over public primary facilities was mainly because of technical quality reasons (poor quality of care, doctor not available, drugs not available, health personnel often absent, no adequate infrastructure).**

**Opare-Addo et al. also found that public hospitals were preferred choices of health facilities because of their perception about offering good quality of care in higher level health facilities. That study also found that negative association of education with utilization of public health centers which agrees with our study findings.**

**After considering all the points, we have inferred that quality assurance of public PHC facilities; by continuous monitoring, assessment and improvement of essential health care infrastructure; improves the utilization of these services for hypertension.**

**Reviewer #3:**

**We thank you for your insightful comments. We responded as below: -**

1. The affiliation of the corresponding author should be consistent. Is it Nay Pyi Taw or Pyin Oo Lwin?

**Yes, we have revised it.**

2. In Figure (2), “twds” in the third predictor variable should be written in full words.

**We have corrected all mistakes.**

3. Abbreviation is needed for LHV in line 220.

**We have added it in abbreviations.**

4. It will be better if you can add the references for "the second reason for lower utilization in urban". For lines 245 and 246.

**It says “urban population; who were experiencing growing urbanization of work and more likely to engage in longer working hours; were dissatisfied with less flexible access and sensitivity to patients’ needs of public sector health care.”**

**A study from Ghana showed that no significant differences between urban and rural, but in multivariate analysis, it showed people were more satisfied with private health facilities in comparing to public/ government health facilities.**

Yaya S, Bishwajit G, Ekholuenetale M, Shah V, Kadio B, Udenigwe O. Urban-rural difference in satisfaction with primary healthcare services in Ghana. BMC Health Services Research. 2017 Nov 25;17(1):776.

**In the revised manuscript, this sentence has not been included in the revision work.**

5. Please provide clarification on whether OOP is needed for both private and public facilities in Myanmar. Because the author’s discussion means to compare two facilities with the same purchasing mechanism for patients' health care. Do patients have to use OOP for all services from public health facilities? Line 249 “When spending OOP payments, people would choose private over public health 250 facilities”

**Thank you for your review.**

**First, what we need to clarify is that public health facilities provide services for free of charge, unlike private facilities where they charge for any services: consultation, diagnostics, medicine and medical supplies. However, patients have to purchase prescribed medicine at retail pharmacies with direct out-of-pocket money, when public sectors have experienced frequent stock-out of essential medicine and medical equipment. So, most OOP is used for self-prescribed medicine and health services at private clinics, described by Than Tun Sein et al.**

**By the time public health sectors cannot provide prescribed medicines, patients would not rather utilize public health facilities if they can access to public hospitals or private facilities (Opare et al). Besides, they would like to be attended by physician, whom they thought to be more skillful than non-physician service providers of public health facilities, as well (Liu et al) because people enjoy additional or higher quality, comprehensive health care rather than basic, essential care.**

**So, if they have to use services spending OOP, they would use at private facilities.**

**Editor Comments:**

**We thank the editor for the useful and relevant comments and we respond as below: -**

- The introduction needs to be restructured and improved. Furthermore, connections between paragraphs are not smooth. Information regarding the important role of primary care services in controlling hypertension is missing.

**Yes, we have restructured the introduction to be smooth as you suggested. So, we have decluttered some sentences and added more information to be complete and concise. We have expressed the role of primary health care (PHC) in controlling hypertension and included sentences about feasibility and effectiveness of hypertension care in PHC, with the added reference.**

- You mentioned 1001 participants, but from how many households? Was there any household replacement?

**Yes, we made household replacement. Total 54 households got replaced for vacant households. We have added the process in methodology session of the manuscript. Of all total households, we got response from 1183 participants of 810 households. Our non-response rate was calculated based on number of participants. So, response rate was 85% (1001 out of 1183). It has been mentioned in revised manuscript.**

- Can you differentiate whether the utilization is at facilities or through outreach according to the data? This information is important for public health interventions. For e.g. many of the participants utilized through outreach, then outreach interventions should be scaled up.

**Unfortunately, no. The information where participants utilized the primary health services for hypertension could not be reported separately.**

**But, according to findings, increased perceived travel cost to the nearest public health facilities decreased the utilization of PHC services provided by those facilities. Bivariate analysis also indicated that presence of public health facilities in their wards or villages increased the utilization of PHC services for hypertension.**

**We have made conclusion from those two points that patients utilized PHC services if the PHC facilities were closer to their home or did not cost them to go the facilities whether it was mobile clinics or through outreach.**

- Did you cover general practitioners (GPs)? Since GPs are important primary care providers in the community, if not, please consider changing the focus to 'utilization of public primary care services' and acknowledge the limitation of not covering GPs.

**No, this study did not cover private PHC facilities including GPs. So, we have discussed it in revision with the added reference.**

**We report changes in reference lists as below: first mentioned lists are removed references and next ones are added references to discuss points raised by all reviewers. We have tried to attempt all comments and discussed accordingly in the revised manuscript.**

**Removed lists:**

**In introduction, we have removed several references about global hypertension and information which makes the manuscript redundant. We have only presented necessary information about the national burden of hypertension. Seven removed references are mentioned below: -**

2.	Mills KT, Stefanescu A, He J. Zhou B, Perel P, Mensah GA, Ezzati M. The global epidemiology of hypertension. Nat Rev Nephrol [Internet]. 2020 Apr [cited 2023 Aug 26];16(4):223–37. Available from: <https://www.ncbi.nlm.nih.gov/pmc/articles/PMC7998524/>

3.	Zhou B, Perel P, Mensah GA, Ezzati M. Global epidemiology, health burden and effective interventions for elevated blood pressure and hypertension. Nat Rev Cardiol. 2021 Nov;18(11):785–802.

4.	Mills KT, Bundy JD, Kelly TN, Reed JE, Kearney PM, Reynolds K, et al. Global Disparities of Hypertension Prevalence and Control. Circulation. 2016 Aug 9;134(6):441–50.

6.	Chaw-Yin Myint, Pavlova M, Groot W. Catastrophic health care expenditure in Myanmar: policy implications in leading progress towards universal health coverage. International Journal for Equity in Health [Internet]. 2019 Jul 30;18(1):118. Available from: <https://doi.org/10.1186/s12939-019-1018-y>

16.	Gulliford M, Figueroa-Munoz J, Morgan M, Hughes D, Gibson B, Beech R, et al. What does “access to health care” mean? J Health Serv Res Policy. 2002 Jul;7(3):186–8.

17.	World Health Organization. World health report 2013: Research for universal health coverage [Internet]. 2013 [cited 2024 Mar 26]. Available from: <https://www.afro.who.int/publications/world-health-report-2013-research-universal-health-coverage>

18.	World Health Organization and the United Nations Children’s Fund (UNICEF). A vision for primary health care in the 21st century: towards universal health coverage and the Sustainable Development Goals [Internet]. Geneva: World Health Organization and the United Nations Children’s Fund (UNICEF); 2018. (Technical Series on Primary Health Care). Available from: <https://www.who.int/docs/default-source/primary-health/vision.pdf>

**In discussion, the prevalence rates of China, India and Nigeria are removed; their references are: -**

28.	Jie W, Kun Y, Wen D. Population and prevalence of hypertension in different regions of China. Zhang L, Defilla S, Chu W, editors. E3S Web Conf [Internet]. 2021 [cited 2024 Mar 27]; 233:02009. Available from: <https://www.e3s-conferences.org/10.1051/e3sconf/202123302009>

32.	Lee J, Wilkens J, Meijer E, Sekher TV, Bloom DE, Hu P. Hypertension awareness, treatment, and control and their association with healthcare access in the middle-aged and older Indian population: A nationwide cohort study. PLoS Med [Internet]. 2022 Jan 4 [cited 2024 Mar 27];19(1):e1003855. Available from: <https://www.ncbi.nlm.nih.gov/pmc/articles/PMC8726460/>

33.	Cic E, Og U, Uw D, Ca A, Sm O, Cc A, et al. Prevalence of Hypertension among adults aged 40 years and above in Ahiazu Mbaise, Imo State, Nigeria. Archives of Community Medicine and Public Health [Internet]. 2018 May 15 [cited 2024 Mar 27];4(1):013–6. Available from: <https://www.peertechzpublications.org/articles/ACMPH-4-134.php>

**During editing for readability, we have also removed an information that seemed redundant in the study. Their references are: -**

37.	Ministry of Helath. Health Facility Assessment: Quality of Maternal, Newborn and Child Health Care | UNICEF Myanmar [Internet]. 2014 Jan [cited 2024 Mar 27]. Available from: <https://www.unicef.org/myanmar/reports/health-facility-assessment-quality-maternal-newborn-and-child-health-care>

38.	World Health Organization. Expanded programme on Immunization (‎EPI)‎ factsheet 2023: Myanmar [Internet]. 2023 [cited 2024 Apr 23]. Available from: <https://www.who.int/publications-detail-redirect/Myanmar-EPI-Factsheet-2023>

44.	Li C, Chen Z, Khan MM. Bypassing primary care facilities: health-seeking behavior of middle age and older adults in China. BMC Health Services Research. 2021 Aug 30;21(1):895.

47.	Mahajan A, Tirakotai W, Masayaanon P. The hidden costs of universal health coverage: solutions from the fight against catastrophic healthcare expenditure in Thailand. BMJ Global Health [Internet]. 2023 Jun 1 [cited 2023 Nov 16];8(6):e011932. Available from: <https://gh.bmj.com/content/8/6/e011932>

50.	Onyeneho NG, Amazigo UV, Njepuome NA, Nwaorgu OC, Okeibunor JC. Perception and utilization of public health services in Southeast Nigeria: Implication for health care in communities with different degrees of urbanization. International Journal for Equity in Health [Internet]. 2016 Jan 20 [cited 2023 Nov 16];15(1):12. Available from: <https://doi.org/10.1186/s12939-016-0294-z>

**Added lists:**

**During modifying introduction, we have added some sentences in the revised manuscript for better connections between paragraphs. Those added references are: -**

5.	Londoño Agudelo E, Battaglioli T, Díaz Piñera A, Rodríguez Salvá A, Smekens T, Achiong Estupiñán F, et al. Effectiveness of a provider and patient-focused intervention to improve hypertension management and control in the primary health care setting in Cuba: a controlled before-after study. BMC Prim Care. 2023 Jan 14;24(1):10. doi: 10.1186/s12875-022-01959-6.

12.	Than Tun Sein, Phone Myint, Nilar Tin, Htay Win, San San Aye, Than Sein. The Republic of the Union of Myanmar Health System Review. Geneva: World Health Organization; 2014. (Health Systems in Transition; vol. 4). Available from: <https://iris.who.int/handle/10665/208211>

14.	World Health Organization. Package of essential noncommunicable (‎PEN)‎ disease interventions for primary health care in low-resource settings. 2010. Available from: <https://www.who.int/publications-detail-redirect/9789241598996>

15.	World Health Organization. Regional Office for South-East Asia. National capacity for prevention and control of NCDs in SEA Region 2019: Country profiles - Myanmar. World Health Organization. Regional Office for South-East Asia.; 2020. Available from: <https://iris.who.int/handle/10665/333801>

**We have added information about how PEN intervention was implemented in public primary health care facilities and not conducted in private facilities, with added references, which is: -**

17.	Win Moh Moh Thit, Kyaw Myint Myat Thein, Khin Thiri Maung, Jat TR, Ko Ko, Kyawt San Lwin, et al. Policy brief-2 Integrating NCD services into primary health care through package of essential non-communicable disease interventions in Myanmar. Yangon: Help Age International; 2017 Oct. Available from: <https://themimu.info/node/114968>

**For added prevalence rates from other South East Asia countries (Indonesia and Sri Lanka), references are: -**

30.	Defianna SR, Santosa A, Probandari A, Dewi FST. Gender Differences in Prevalence and Risk Factors for Hypertension among Adult Populations: A Cross-Sectional Study in Indonesia. International Journal of Environmental Research and Public Health. 2021 Jun;18(12). doi: 10.3390/ijerph18126259.

31.	Rannan-Eliya RP, Wijemunige N, Perera P, Kapuge Y, Gunawardana N, Sigera C, et al. Prevalence and Associations of Hypertension in Sri Lankan Adults: Estimates from the SLHAS 2018–19 Survey Using JNC7 and ACC/AHA 2017 Guidelines. Global Heart. 2022 ;17(1). doi: 10.5334/gh.1135.

**We have discussed about low participation of male in cross-sectional study with these two added references in revision. They are: -**

30.	Defianna SR, Santosa A, Probandari A, Dewi FST. Gender Differences in Prevalence and Risk Factors for Hypertension among Adult Populations: A Cross-Sectional Study in Indonesia. International Journal of Environmental Research and Public Health [Internet]. 2021 Jun [cited 2024 Aug 20];18(12). Available from: <https://www.ncbi.nlm.nih.gov/pmc/articles/PMC8296037/>

32.	Mohanty P, Patnaik L, Nayak G, Dutta A. Gender difference in prevalence of hypertension among Indians across various age-groups: a report from multiple nationally representative samples. BMC Public Health. 2022 Aug 10;22(1):1524. doi: 10.1186/s12889-022-13949-5.

**We have added a reference to support the idea of how quality assurance of health facilities improve utilization of health services. Added reference in revision is: -**

38.	Kujawski SA, Leslie HH, Prabhakaran D, Singh K, Kruk ME. Reasons for low utilisation of public facilities among households with hypertension: analysis of a population-based survey in India. BMJ Glob Health. 2018;3(6):e001002. doi: 10.1136/bmjgh-2018-001002.

**We have discussed how urban residents choose health facilities based on their conveniences in the revised manuscript. The added references are as below: -**

41.	Moturi AK, Suiyanka L, Mumo E, Snow RW, Okiro EA, Macharia PM. Geographic accessibility to public and private health facilities in Kenya in 2021: An updated geocoded inventory and spatial analysis. Front Public Health. 2022 Nov 3;10. doi: 10.3389/fpubh.2022.1002975.

42.	Aung M, Oo W, Lwin K, Maung Maung T. Health services utilization and self-reported acute illnesses among urban families in Thanlyin Township, Yangon Region, Myanmar. International Journal of Health Sciences and Research. 2016 Jan 6;6:36–42.

**We have added discussion about how good perception and high level education influence on utilization of public PHC facilities by using below mentioned references: -**

43.	Karmakar N, Nag K, Saha I, Parthasarathi R, Patra M, Sinha R. Awareness, treatment, and control of hypertension among adult population in a rural community of Singur block, Hooghly District, West Bengal. J Educ Health Promot. 2018 Oct 29;7:134. doi: 10.4103/jehp.jehp_164_18.

44.	Shiraly R, Jeihooni AK, Shirazi RB. Perception of risk of hypertension related complications and adherence to antihypertensive drugs: a primary healthcare based cross-sectional study. BMC Primary Care. 2022;23. doi: 10.1186/s12875-022-01918-1.

**As for operation hours of PHC facilities, we have discussed it in the discussion section with added references in the revised manuscript. They are: -**

49.	Zhang W, Ung COL, Lin G, Liu J, Li W, Hu H, et al. Factors Contributing to Patients’ Preferences for Primary Health Care Institutions in China: A Qualitative Study. Front Public Health. 2020 Aug 18;8:414. doi: 10.3389/fpubh.2020.00414.

50.	Alqossayir FM, Alkhowailed MS, Alammar AY, Alsaeed AA, Alamri YY, Rasheed Z. Factors associated with patients bypassing primary healthcare centres in Qassim Region, KSA. J Taibah Univ Med Sci. 2021 Aug 21;16(6):900–5. doi: 10.1016/j.jtumed.2021.07.012.

**Furthermore, we have submitted our protocol in protocol.io and got**

**DOI: dx.doi.org/10.17504/protocols.io.8epv5rk8dg1b/v1 (Private link for reviewers:**

[**https://www.protocols.io/private/092FFA5A645611EF96470A58A9FEAC02**](https://www.protocols.io/private/092FFA5A645611EF96470A58A9FEAC02) **to be removed before publication.)**

**If there are further points that need addressing, we are happy to revise the manuscript accordingly.**

**Yours sincerely**

**May Sabai Soe**

**On behalf of all co-authors**
