# Supplementary material for: Prevalence of hypertension and factors associated with the utilization of primary health care services for hypertension among hypertensive population aged 40 years and above in Pyin Oo Lwin Township, Myanmar
Source: PLoS One. 2024 Oct 16;19(10):e0312186. doi: 10.1371/journal.pone.0312186 (PMC11482684; doi:10.1371/journal.pone.0312186)
Supplement: Attachment — Submitted filename: Reviewer comments.docx [file pone.0312186.s004.docx]

In Financial Disclosure, it is mentioned that “The author(s) received no specific funding for this work”. However, the authors acknowledged the IR grant committee of Department of Medical Research, Ministry of Health. Could you please clarify whether this study was funded by the grant from IR grant committee of Department of Medical Research, Ministry of Health? If not, could you explain how the expenses for this study were covered.

I would like to suggest a through proofread. Some sentences are lengthy and could be rephrased for better clarity and readability.

The abstract should serve as a standalone summary, including all essential details for comprehension without referring to the full manuscript. In this abstract, methods need to be elaborated further, and results and conclusion should be aligned.

Line 29: Please specify the year in which stroke was the first leading cause of death in Myanmar

Line 42: Please correct the grammar for “PHC-oriented research were needed”

In Introduction section, it would be better to explain the reasons for choosing Pyin Oo Lwin Township and the population aged 40 years and above. Additionally, please clarify whether PHC services users need to pay for consultation fees or medicine.

Line 51-59: it is better to provide descriptions of other PHC services providers in study site such as GP clinics, or outpatient departments which are not under the township public health department and should explain the reasons why these are not included as PHC services providers in this study.

Line 71-79: It would be helpful to provide description of the location of the 7 villages and 3 wards.

Line 98: “Data was collected” should be “Data were collected”

Line 104: “two-parts” should be “two parts”

Line 161: Please clarify “not aware of their statuses”. I assume “not aware of their hypertension”.

Line 168: “Table (1) showed” should be “Table (1) shows”

Line 173: Could you please clarify the rationale for using 150,000 MMK as the cutoff point?

Line 181: “Shown in figure (2)” should be “As shown in figure (2)”.

Line 185-186: Please clarify high perception and low perception.

In Results section, it would be better to include relevant findings from bivariate analysis. Additionally, important negative findings-factors not associated with utilization of PHC services-should also be described and discussed in the main manuscript.

Line 195: “Age composition” should be “The age composition”

Line 199: “30-79Years” should be “30-79 years”

Line 207: Can you please spell out “SEAR”

Line 220: Please spell out LHV

Line 226: I would like to suggest using “However” instead of “But” at the beginning of the sentence.

Line 248-249: Could you please elaborate more on “Consequently, urban residents choose health facilities based on their conveniences.”

Line 270: I would like to suggest “economic inaccessibility” instead of “economic accessibility”

In Discussion section: It would be better to explore the possible reasons behind the fact that over two third of the participants were female, as well as operation hours of the PHC service centers. Discussing the implication of these findings and comparisons with other studies could be beneficial. For instance, consider addressing how these differences or similarities affect our understanding of hypertension prevalence, management and PHC utilization in your study population. Furthermore, discussing potential strategies, policy recommendation, and areas for further research to improve the situation would be valuable.

Line 296-298: Could you please provide more details on how the findings from this study support the idea that quality assurance of PHC services improves the utilization of these services for hypertension.
